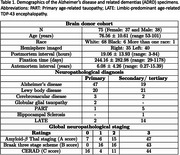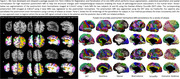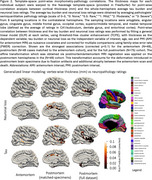# High‐resolution postmortem 7 tesla MRI yields localized atrophy measures that are more sensitive to tau pathology and neuronal loss in Alzheimer's disease than corresponding measures on antemortem 3 tesla MRI

**DOI:** 10.1002/alz70856_098995

**Published:** 2025-12-24

**Authors:** Pulkit Khandelwal, Michael Tran Duong, Lisa M Levorse, Sydney A. Lim, Amanda E Denning, Nathaniel Gauthier, Ved Shenoy, Winifred Trotman, Ranjit Ittyerah, Alejandra Bahena, Theresa Schuck, Marianna Gabrielyan, Karthik Prabhakaran, Daniel T Ohm, Gabor Mizsei, John L. Robinson, Laura E.M. Wisse, John A. Detre, Eddie B. Lee, David J. Irwin, Corey T. McMillan, M. Dylan Tisdall, Sandhitsu R. Das, David A. Wolk, Paul A. Yushkevich

**Affiliations:** ^1^ University of Pennsylvania, Philadelphia, PA, USA; ^2^ Department of Pathology and Laboratory Medicine, Institute on Aging and Center for Neurodegenerative Disease Research, The Perelman School of Medicine at the University of Pennsylvania, Philadelphia, PA, USA; ^3^ Digital Neuropathology Laboratory, University of Pennsylvania, Philadelphia, PA, USA; ^4^ Lund University, Lund, Sweden; ^5^ Department of Pathology & Laboratory Medicine, Perelman School of Medicine, University of Pennsylvania, Philadelphia, PA, USA; ^6^ Penn FTD Center, University of Pennsylvania, Philadelphia, PA, USA; ^7^ Penn Image Computing and Science Laboratory (PICSL), University of Pennsylvania, Philadelphia, PA, USA; ^8^ Penn Memory Center, University of Pennsylvania, Philadelphia, PA, USA; ^9^ Penn Alzheimer's Disease Research Center, University of Pennsylvania, Philadelphia, PA, USA

## Abstract

**Background:**

Postmortem MRI has opened‐up avenues to study brain structure at ultra high‐resolution revealing details not possible to observe with in vivo MRI. Here, we present a novel package (purple‐mri) which performs tissue segmentation, anatomical parcellation and spatial normalization of postmortem MRI. Additionally, we provide a framework to perform point‐wise surface‐based group‐level studies linking morphometry/histopathology in common coordinate system for postmortem MRI.

**Method:**

We developed a joint voxel‐ and surface‐based pipeline combining deep learning with classical techniques for topology correction, cortical modeling, inflation, and registration for accurate parcellation of postmortem cerebral hemispheres (Figure 1 Khandelwal et al. 2024). Furthermore, using the GM/WM segmentations derived from postmortem hemisphere and FreeSurfer‐processed antemortem MRI, we performed deformable image registration between the ante‐ and postmortem MRI for each brain specimen. To demonstrate the utility of purple‐mri, point‐wise analysis was performed to correlate thickness (mm) with tau and neuronal loss distribution in corresponding specimens (*N* = 49) of postmortem (7T at 0.3mm^3^) and antemortem (3T at 0.8mm^3^) MRI (Table 1) within the AD continuum diagnosis. An additional 26 postmortem 7T scans without corresponding antemortem scans were included in some analyses. The semi‐quantitative average tau and neuronal loss ratings were derived from histopathological examination across the brain. All analyses include age, sex, and postmortem (or antemortem) interval as covariates.

**Result:**

Our method parcellates postmortem brain hemisphere using a variety of brain atlases even in areas with low contrast (anterior/posterior regions), profound imaging artifacts and severely atrophied brains (Figure 1). Our registration pipeline provides one‐to‐one correspondence between the two modalities. For thickness/pathology associations (Figure 2), small sparse significant clusters only in superior temporal cortex and precuneus in antemortem MRI (*N* = 49) were observed. However, postmortem MRI showed much stronger associations across large clusters in temporal, entorhinal cortex, and cingulate for both the matched cases (*N* = 49) and the full cohort (*N* = 75), regions implicated in ADRD.

**Conclusion:**

Purple‐mri paves the way for large‐scale postmortem image analysis. Stronger associations between thickness and average tau burden/neuronal loss than antemortem MRI suggests that our pipeline could inform the development of more precise and sensitive invivo biomarkers by mapping information from postmortem to antemortem MRI in a common reference coordinate system.